# Hydro-Geochemistry of the River Water in the Jiulongjiang River Basin, Southeast China: Implications of Anthropogenic Inputs and Chemical Weathering

**DOI:** 10.3390/ijerph16030440

**Published:** 2019-02-02

**Authors:** Xiaoqiang Li, Guilin Han, Man Liu, Kunhua Yang, Jinke Liu

**Affiliations:** Institute of Earth Sciences, China University of Geosciences (Beijing), Beijing 100083, China; xiaoqli@cugb.edu.cn (X.L.); lman@cugb.edu.cn (M.L.); kunhuayang@cugb.edu.cn (K.Y.); liujinke@cugb.edu.cn (J.L.)

**Keywords:** chemical weathering, major ions, human activities, Jiulongjiang River, southeast China

## Abstract

This study focuses on the chemical weathering process under the influence of human activities in the Jiulongjiang River basin, which is the most developed and heavily polluted area in southeast China. The average total dissolved solid (TDS) of the river water is 116.6 mg/L and total cation concentration (${\mathrm{TZ}}^{+}$) is 1.5 meq/L. Calcium and ${\mathrm{HCO}}_{3}^{-}$ followed by ${\mathrm{Na}}^{+}$ and ${\mathrm{SO}}_{4}^{2-}$ constitute the main species in river waters. A mass balance based on cations calculation indicated that the silicate weathering (43.3%), carbonate weathering (30.7%), atmospheric (15.6%) and anthropogenic inputs (10.4%) are four reservoirs contributing to the dissolved load. Silicates (SCW) and carbonates (CCW) chemical weathering rates are calculated to be approximately 53.2 ton/km^2^/a and 15.0 ton/km^2^/a, respectively. When sulfuric and nitric acid from rainfall affected by human activities are involved in the weathering process, the actual atmospheric ${\mathrm{CO}}_{2}$ consumption rates are estimated at 3.7 × 10^5^ mol/km^2^/a for silicate weathering and 2.2 × 10^5^ mol/km^2^/a for carbonate weathering. An overestimated carbon sink (17.4 Gg C/a) is about 27.0% of the ${\mathrm{CO}}_{2}$ consumption flux via silicate weathering in the Jiulongjiang River basin, this result shows the strong effects of anthropogenic factors on atmospheric ${\mathrm{CO}}_{2}$ level and current and future climate change of earth.

## 1. Introduction

Chemical weathering of rocks is part of the exogenous cycle, in which periodic migration of elements occurs among lithosphere, atmosphere, biosphere and hydrosphere [1,2,3,4,5,6]. The latest report (Global Carbon Budge 2017) shows that the present-day concentration of carbon dioxide is over 400 ppm in the atmosphere [7], consequently, some natural hazards such as continuous heatwaves and extreme rainfall are likely to become more common because of the accumulation of greenhouse gases (${\mathrm{CO}}_{2}$). However, the earth’s exogenic cycle resulted in many different interpretations of the chemical weathering of rocks on the earth surface and the global carbon cycle [8]. One of the important hypotheses is that climate may be a main controlling factor in silicate weathering as a net carbon sink in long-term continental denudation, because higher temperatures and runoff can accelerate the weathering process [9,10]. Although the relationship between chemical weathering and carbon cycle has been investigated in most of rivers in the world, remaining doubts continue to influence our understanding of connections among rock weathering, climate and the carbon cycle due to huge difference in geological environment, which leads to great uncertainty to quantitative analysis of the global carbon cycle [1,4,6,8,10].

As an important transport pathway, rivers reflect various natural and anthropogenic processes in watershed, and also carry and transport the weathering products and anthropogenic pollutants from continents to the oceans [11,12]. Therefore, many scholars have focused on rivers system, using material fluxes of rivers to estimate chemical weathering rates and atmospheric ${\mathrm{CO}}_{2}$ consumption flux in small basins and global scale [13,14,15,16,17]. Since the industrial revolution, increasing the anthropogenic interference with natural systems has sharply influenced water quality and natural weathering processes [18,19,20,21]. These pollutions from agriculture or industry often mask the real signals of rock weathering to some extent. For example, strong inorganic acid deposition (mainly sulfuric acid or nitric acid) originated from human activities could partly mask the real signal of carbonic acid in the process of continental denudation [14,22]. For example, in the major rivers of the South Korean, the actual atmospheric CO_2_ consumption is reduced to 64.9% of the value estimated when carbonic acid provides all the protons in the weathering reactions [23]. In this case, chemical weathering of silicate will lead to a source of ${\mathrm{CO}}_{2}$ in the atmosphere rather than a sink, because a fraction of ${\mathrm{CO}}_{2}$ is released to the atmosphere when calcite or dolomite precipitation occurs in the oceans [22]. The nonnegligible role of acid deposition during the weathering process has been reported in some river basins [14,23,24,25]. Compared to carbonate weathering, the silicate weathering reaction is more easily accelerated by strong acids from rainfall owing to their relatively weak buffeting capability [26,27].

Jiulongjiang River basin (JRB) is a coastal basin covered by Mesozoic granites of the Fujian province. Furthermore, it is also the most severely affected by acid deposition in China. Therefore, this is a suitable area in which the influence of acid rain can be studied. Although previous research had assessed the chemical composition of river water in the JRB [27], all river water samples were only collected and analyzed in the summer of 2010. In this paper, systematic analysis of the Jiulongjiang River (JR) have been implemented to show the spatiotemporal variation characteristics of major ions and material flux in 2017–2018. Furthermore, to assess current and future environmental quality, the chemical composition and hydrologic data of the study area are used to calculate the rates of chemical weathering of rocks and associated atmospheric ${\mathrm{CO}}_{2}$ consumption (carbon sink) under the effects of acid deposition.

## 2. Materials and Methods

### 2.1. Study Site

**Figure 1:**
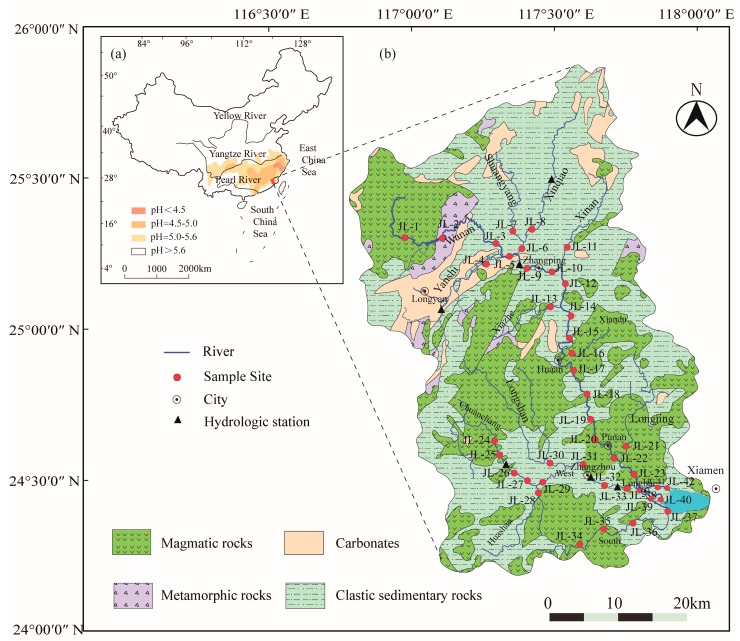
(**a**) The People’s Republic of China with the location of Jiulongjiang River basin (JRB). (**b**) Simplified geological map of the JRB with sampling sites. JL-1 to JL-42 represent the number of 42 water sampling sites in the Jiulongjiang River (JR).

The study site is located in the southeast of China (24°18′ to 25°88′ N latitude, 116°78′ to 118°03′ E longitude) (Figure 1). The JRB, with a drainage area of 1.41 × 10^4^ km^2^ and an average annual discharge of 1.45 × 10^10^ $m^{3}/a$, is a typical medium-sized subtropical coastal watershed [28]. The JR consists of mainstream (North River) and major tributaries (West, South, Yanshi, Xinqiao, Shuangyang, Xinan, Longjing, Chuanchang, Huashan and Longshan River) with an area over 500 km^2^. Average annual temperature in the JRB varies from 19.9 °C to 21.1 °C [27]. Because of the influence of a seasonal monsoonal climate, approximately 75% of the total water discharge occurs during summer from April to September [29]. Due to the impacts of climate and NE-trending fault zone, the spatial and temporal differences of precipitation are significant, and the annual precipitation varies between 1400 and 1800 mm (average, 1684 mm) with an increasing trend from estuarine to mountainous environments [30]. The North River (244 km) occupies an area of 8490 km^2^, and it originated in the mountains of the northwest part of the basin. The West river (156 km) is the largest tributary with an area of 3737 km^2^, originating in the southern Shifang mountain of Longyan city. The JRB, located in the South China Block, is an important tectonic belt where the Eurasian plate interacts with the Pacific plate. The JRB consists of silicate and carbonate rocks, no obvious evaporite distribution in the study area. A large number of magmatic activities took place in the Mesozoic, and the proportions of magmatic rocks is over 60% [27]. The outcrop intrusive rocks are mainly biotite granite and granodiorite. The sedimentary rocks in the basin cover a large area, including sandstone as well as carbonate rocks such as limestone and dolomite mainly distributed in the upper reaches of the North river. The basin is rich in mineral resources, for instance, a large deposit bearing Fe hosted in the interlayer fracture zone between carbonates and clastic rocks in Longyan city.

### 2.2. Water Sampling and Analysis

A total of 84 river water samples (forty-two sample sites) from the JR were collected in acid-washed containers in June 2017 and January 2018 which correspond to summer and winter, respectively (sample number and locations are shown in Figure 1). At the sample points, temperature (T), electrical conductivity (EC) and pH of the water samples were measured using a YSI ProPlus (YSI Inc., Yellow Springs, OH, USA), and the alkalinity was determined by methyl red and bromocresol green indicator with pure HCl titration. The pH probe was calibrated at 4, 7 and 10, the EC probe at 12.7 before sampling. All water samples passed pre-washed 0.22 μm cellulose acetate member (Millipore). Filtered water samples were stored in acid-washed HDPE bottles for analysis cations after acidification to pH < 2 with pure HCl or HNO_3_. Dissolved silica (SiO_2_) was measured by spectrophotometry. Major cations (${\mathrm{Mg}}^{2+}$, ${\mathrm{Ca}}^{2+}$, $K^{+}$ and ${\mathrm{Na}}^{+}$) were measured by using ICP-OES (Optima 5300DV, PerkinElmer Inc., Waltham, MA, USA) with a precision of ±3% in the Institute of Geographic Sciences and Nature Resources Research, Chinese Academy of Sciences (CAS). Anions (${\mathrm{SO}}_{4}^{2-}$, ${\mathrm{Cl}}^{-}$ and ${\mathrm{NO}}_{3}^{-}$) were analyzed in filtered unacidified samples by ionic chromatography (Dionex 1100, USA) with a precision of ±5%. Quality control procedures, ions reference materials (GSB, China) were employed to check the accuracy and precision of the test results.

### 2.3. Data Processing

The simplified geology map in this study was based on the “Geological Map of Fujian Province” (1:500,000). The physical–chemical parameters and major ions were treated by Microsoft Excel (Microsoft, Redmond, WA, USA). Figures were drawn using Sigma Plot 12.5 software (Systat Software Inc., San Jose, CA, USA) and Adobe Illustrator CC 2015.3 (Adobe Inc., San Jose, CA, USA). Other equations used in this article are described in the following sections.

**Figure 2:**
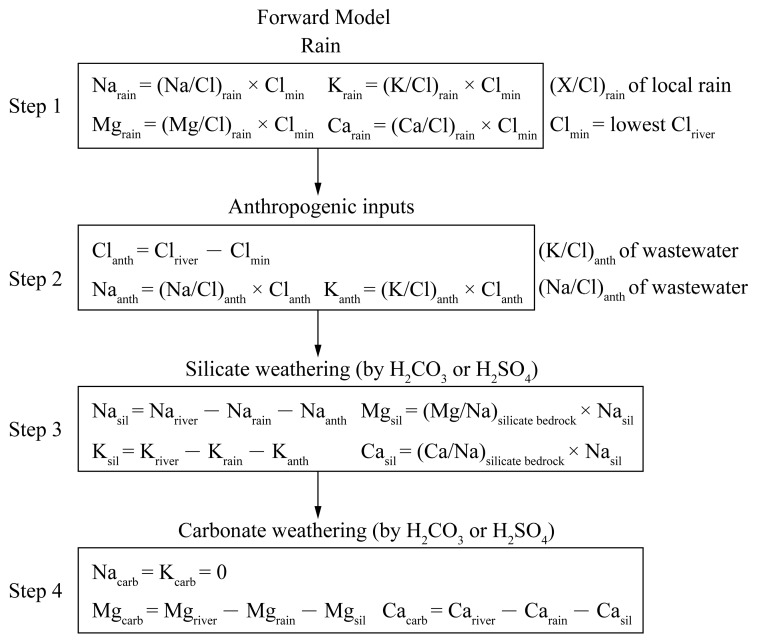
An outline of the forward model calculations for cations ($K^{+}$, ${\mathrm{Na}}^{+}$, ${\mathrm{Ca}}^{2+}$ and ${\mathrm{Mg}}^{2+}$) from four major reservoirs (rain, anthropogenic inputs, silicate and carbonate weathering) in the JRB.

A forward model (Figure 2) was based on mass budget equations of cations ($K^{+}$, ${\mathrm{Na}}^{+}$, ${\mathrm{Ca}}^{2+}$ and ${\mathrm{Mg}}^{2+}$) from four major reservoirs (rain, anthropogenic inputs, silicate and carbonate weathering) in the JRB [1]. In step 1, we assumed that the water sample with lowest Cl concentration got all Cl from rain. According (X/Cl)_rain_ of local rain, we can calculate the input from rain, then the excess Cl corrected for rain could be attributed to the human activities [27]. In step 2, wastewater is used as an indicator of human activities. Here we use only sodium and potassium as a proxy for anthropogenic inputs, because the amount of calcium and magnesium from human activities is negligible compared with those from rocks [15]. In step 3, calcium and magnesium from silicates were estimated by using average (Ca/Na)_silicate bedrock_ and (Mg/Na)_silicate bedrock_ complied from literatures containing geochemical data. In step 4, we calculated the input from carbonate weathering by subtracting the rain and silicate contributions from the total dissolved Ca and Mg in rivers.

## 3. Results

### 3.1. Physical–Chemical Parameters

The field physical and chemical parameters of river water samples are presented in Table S1. Most of the water samples are slightly acidic to alkaline from 6.3 to 8.8 in the JR, and the average value is 7.2. The water temperature measured at sampling sites is from 21.1 °C to 31.9 °C in summer (average, 26.2 °C) and from 11.0 °C to 22.7 °C in winter (average, 16.4 °C). The chemical compositions of the JR water are highly variable, and the concentrations of total dissolved solid (TDS = ${\mathrm{Ca}}^{2+}$ + ${\mathrm{Mg}}^{2+}$ + ${\mathrm{Na}}^{+}$ + $K^{+}$ + ${\mathrm{Cl}}^{-}$ + ${\mathrm{NO}}_{3}^{-}$ + $0{.5\mathrm{HCO}}_{3}^{-}$ + ${\mathrm{SO}}_{4}^{2-}$ + ${\mathrm{SiO}}_{2}$) are 23.2 (JL-1) to 700.7 mg/L (JL-41) in summer and 23.8 (JL-1) to 10,056.7 mg/L (JL-37) in winter. It is worth noting that some samples (JL-37, JL-39 to JL-41 in winter) have unusually values of TDS (≥5000 mg/L) result from mixing of the seawater in the estuary. TDS values in the mainstream increase slowly downstream owing to the limited contribution from the tributaries. The average TDS values of the JR is 99.2 mg/L in summer and 133.9 mg/L (exclude the JL-37, JL-39 to JL-42, the same below) in winter with an annual average of 120.8 mg/L. EC has similar characteristics with the TDS. The EC varies from 17.8 (JL-1) to 1482.0 μs/cm (JL-41) in summer and from 26.8 (JL-1) to 1603.0 μs/cm (JL-4) in winter. The total cationic charges (${\mathrm{TZ}}^{+}$ = 2${\mathrm{Ca}}^{2+}$ + 2${\mathrm{Mg}}^{2+}$ + ${\mathrm{Na}}^{+}$ + $K^{+}$, in meq/L) are from 0.2 to 11.7 meq/L in summer and from 0.2 to 14.3 meq/L in winter and a weighted average value is 1.5 meq/L, and it is higher than the mean value (TZ^+^ = 1.125 meq/L) of world rivers [31]. In 84 water samples analyzed, only 9 water samples (in winter) displayed a normalized inorganic charge balance (NICB = (${\mathrm{TZ}}^{+}$ − ${\mathrm{TZ}}^{-}$)/${\mathrm{TZ}}^{-}$ × 100) more than ±10%, where ${\mathrm{TZ}}^{-}$ = ${\mathrm{Cl}}^{-}$ + ${\mathrm{NO}}_{3}^{-}$ + ${\mathrm{HCO}}_{3}^{-}$ + 2${\mathrm{SO}}_{4}^{2-}$ in meq/L.

### 3.2. Temporal and Spatial Distribution of Major Ions and Dissolved Silica

**Figure 3:**
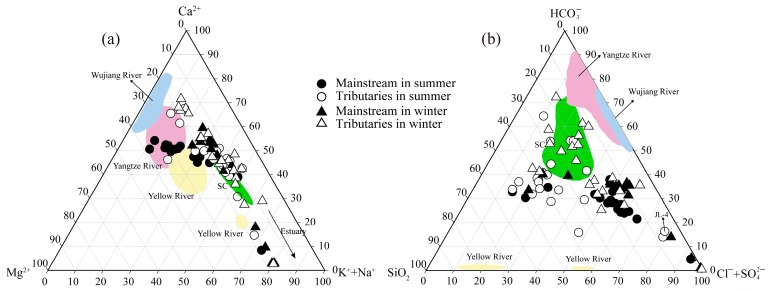
Ternary diagrams of (**a**) cations, (**b**) anions and dissolved silica in the JR. Some large rivers are also shown in China. Data sources: Wujiang River [32], Yellow River [13], Yangtze River [15] and major rivers on the southeast coastal in China (SC) [27].

The proportion of major ions is shown in the cation and anion ternary diagrams (Figure 3). For all of the samples, ${\mathrm{Ca}}^{2+}$ is the major cation with concentrations ranging from 1.5 (JL-1) to 43.6 mg/L (JL-4), accounting for 47.8% (average percentage, in meq/L) of all cations in summer and 47.9% in winter. The highest proportion occurs in the Xinan River (67.5% in summer and 71.8% in winter) which drainages carbonate rocks relief. The next most common cations are ${\mathrm{Na}}^{+}$ (1.5 to 231.0 mg/L) and ${\mathrm{Mg}}^{2+}$ (0.3 to 28.6 mg/L), regardless of sampling season, accounting for average 24.3% and 17.8% of ${\mathrm{TZ}}^{+}$, respectively. The ${\mathrm{Ca}}^{2+}$/${\mathrm{Na}}^{+}$ and ${\mathrm{Mg}}^{2+}$/${\mathrm{Na}}^{+}$ ratios present distinct values in river waters, ranging from 0.02 to 4.4 and from 0.07 to 2.4, respectively. The highest values are observed in the Xinan River and North River (Zhangping), respectively. Potassium takes up the very small proportion (about 8.3%) of the cations. For anions, ${\mathrm{HCO}}_{3}^{-}$ is the dominant anion in the JR, with concentrations ranging from 7.5 (JL-1) to 84.9 mg/L (JL-4). On average, it accounts for 8.9% to 83.5% of the total anions (average, 42.0%). Another common anion is ${\mathrm{SO}}_{4}^{2-}$ with concentrations ranging from 1.4 (JL-1) to 92.3 mg/L (JL-4), and accounted for 6.9% to 64.7% of the total anion (average, 26.8%). The proportions of ${\mathrm{Cl}}^{-}$ (0.9 to 424.2 mg/L) and ${\mathrm{NO}}_{3}^{-}$ (0.9 to 69.0 mg/L) account approximately 15.5% and 15.7% of the total anions, respectively. The concentration of dissolved silica (8.7 to 27.8 mg/L) is higher compared to other rivers, this characteristic coupled with high ${\mathrm{Cl}}^{-}$ + ${\mathrm{SO}}_{4}^{2-}$ makes most water samples clustered toward the center of cations ternary diagrams (Figure 3b).

## 4. Discussion

Rivers are the major transport paths of dissolved and solid matter. It is reported that a large amount of dissolved substances (about 3.8 Gt) are annually transferred to the oceans by rivers [33]. In general, the dissolved substances of the river waters are mainly the products of weathered rocks. However, the contribution of atmospheric and anthropogenic inputs is significant in some coastal areas due to high population density. So, it is necessary to correct the dissolved load from atmospheric (mainly wet deposition) and anthropogenic inputs when calculating the contribution of rock weathering to solute [34].

### 4.1. Atmospheric Deposition

In atmospheric deposition, $K^{+}$, ${\mathrm{Ca}}^{2+}$ and ${\mathrm{Mg}}^{2+}$ primarily derived from terrestrial dust, while ${\mathrm{SO}}_{4}^{2-}$ and ${\mathrm{NO}}_{3}^{-}$ are considered to be mainly from human activities, for instance, coal combustion [35]. For ${\mathrm{Na}}^{+}$ and ${\mathrm{Cl}}^{-}$, ocean aerosols would make up a large proportion. As shown in many studies, the chlorine-based method is used to evaluate the inputs of atmospheric deposition into rivers because of its conservative properties [36,37]. For an element X in river water, the non-cyclic concentration is $X_{\mathrm{river}}^{*}$:

(1) $$X_{\mathrm{river}}^{*}=X_{\mathrm{river}}-{(X/\mathrm{Cl})}_{\mathrm{rain}}\times {\mathrm{Cl}}_{\min}$$

In Equations (1), $X_{\mathrm{river}}^{*}$ is the corrected ion content derived from rainwater, $X_{\mathrm{river}}$ is original concentration of element X in river waters without correction. ${\left(X/\mathrm{Cl}\right)}_{\mathrm{rain}}$ is the ratio of any element (X) to ${\mathrm{Cl}}^{-}$ in the rain, ${\mathrm{Cl}}_{\min}$ stands for the lowest Cl content in river waters and assumed that ${\mathrm{Cl}}_{\min}$ is entirely derived from rainwater without the contribution of evaporites.

**Figure 4:**
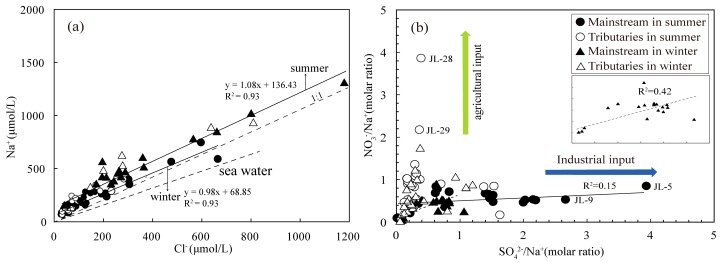
(**a**) Relationship between ${\mathrm{Na}}^{+}$ and ${\mathrm{Cl}}^{-}$ in the JR. Data of sea water (Na/Cl = 0.89) is from Hagedorn and Cartwright [43]. (**b**) Relationship between ${\mathrm{NO}}_{3}^{-}$/${\mathrm{Na}}^{+}$ and ${\mathrm{SO}}_{4}^{2-}$/${\mathrm{Na}}^{+}$ for river waters in the JR.

The JR is located in east China’s acid rain zone with an annual average pH of 5.4 (Table 1). Moreover, Yu, et al. [41] studied the possible source of ${\mathrm{SO}}_{4}^{2-}$ and ${\mathrm{NO}}_{3}^{-}$ from rainfall in Xiamen, and the results showed that the contribution of local anthropogenic pollution source was up to 54.2%. On the whole, the major anion of rain is ${\mathrm{SO}}_{4}^{2-}$, with an average concentration of 2.8 mg/L. In the data of rainwater, the average value of ${\mathrm{SO}}_{4}^{2-}$/${\mathrm{Na}}^{+}$ is 1.5, this implies that sulfate ion is enriched compared to seawater (${\mathrm{SO}}_{4}^{2-}$/${\mathrm{Na}}^{+}$ = 0.06) [42]. The ratio of ${\mathrm{Na}}^{+}$/${\mathrm{Cl}}^{-}$ in river waters is slightly higher in winter than in summer, and Na markedly exceeded Cl owing to anthropogenic inputs (Figure 4a). 

The content of ${\mathrm{Cl}}^{-}$ in the headwater (Wanan, JL-1) is the lowest about 0.9 mg/L in summer and 1.1 mg/L in winter. Because this area is located remote from the city and estuary, so we assumed that all the ${\mathrm{Cl}}^{-}$ in JL-1 come from rainfall. On this basis, the contribution of rainwater for cations is 17.6% (4.8%–63.8%) in summer and 14.1% (4.4%–70.8%) in winter. For anions, about half of the ${\mathrm{SO}}_{4}^{2-}$ comes from the rain (55.4% in summer and 49.5% in winter) and about 32% of ${\mathrm{NO}}_{3}^{-}$ from atmospheric inputs.

### 4.2. Anthropogenic Disturbance

The products of human activities could enter the rivers through precipitation, agricultural activities and industrial wastewater [44]. With the rapid development of industry and agriculture in the recent ten years, the water pollution of JR is a serious issue [45]. In the river, $K^{+}$, ${\mathrm{Na}}^{+}$, ${\mathrm{Cl}}^{-}$, ${\mathrm{NO}}_{3}^{-}$ and ${\mathrm{SO}}_{4}^{2-}$ are usually related to human activities, therefore these ions have been used as tracer in identifying the degree of anthropogenic contribution [27]. In this study, there is a significant increase in the concentration of ${\mathrm{NO}}_{3}^{-}$ and $K^{+}$ in the lower reaches of Huashan River (JL-28) and this increase may be closely connected to agriculture production (fertilizers and herbicide) [43], while the concentration of chlorine may be related to urban wastewater, for example, Longyan city (JL-4, 23.58 mg/L in summer and 21.35 mg/L in winter). Riverine ${\mathrm{SO}}_{4}^{2-}$ is generally derived from several sources such as oxidation of sulfide minerals, acid rain and dissolution of gypsum [1]. Highest concentration of ${\mathrm{SO}}_{4}^{2-}$ appears in Yanshi River (JL-4, 92.3 mg/L in summer and 71.5 mg/L in winter). For JL-4, after correcting for precipitation, remaining sulfate ions (more than 90% of sulfate ions) may come from the sulfide minerals in iron mine. Moreover, no linear correlation is observed between ${\mathrm{SO}}_{4}^{2-}$/${\mathrm{Na}}^{+}$ and ${\mathrm{NO}}_{3}^{-}$/${\mathrm{Na}}^{+}$ (R^2^ = 0.15 in summer and R^2^ = 0.42 in winter) in the mainstream (Figure 4b), this might suggest that ${\mathrm{SO}}_{4}^{2-}$ and ${\mathrm{NO}}_{3}^{-}$ could have different sources. The peak value of ${\mathrm{NO}}_{3}^{-}$/${\mathrm{Na}}^{+}$ appears in Huashan River (JL-28) draining a typical agricultural zone, while some samples have a relatively high ${\mathrm{SO}}_{4}^{2-}$/${\mathrm{Na}}^{+}$ (>2) and low ${\mathrm{NO}}_{3}^{-}$/${\mathrm{Na}}^{+}$ (<1), this may suggest that these samples are more subjected to communal wastewater. After deducting the contribution of atmospheric precipitation, it is estimated about 68.0% of ${\mathrm{NO}}_{3}^{-}$ and 68.7 of ${\mathrm{Cl}}^{-}$ originate from anthropogenic inputs (mainly wastewater from agricultural production) in the JR. For $K^{+}$ and ${\mathrm{Na}}^{+}$, a set of reference value (($K^{+}{/\mathrm{Cl}}^{-}$)_anth_ = 0.3 and (${\mathrm{Na}}^{+}{/\mathrm{Cl}}^{-}$)_anth_ = 0.2 in wastewater) is used to calculation the contribution from anthropogenic inputs [15]. The results show that (${\mathrm{Na}}^{+}$_anth_ + $K^{+}$_anth_) and ${\mathrm{Cl}}^{-}$_anth_ from anthropogenic inputs account for 9.7% and 68.7% of dissolved cations, respectively.

### 4.3. Rock Chemical Weathering Inputs

The exhumation of rocks from the upper continental crust implies that they have to go through an adjustment from higher temperature and pressure to lower temperature and pressure [8]. Therefore, a series of reactions will occur, such as hydration, dissolution, hydrolysis and carbonation. This adjustment may be relatively fast for evaporates and carbonates or relatively slow for some silicate minerals, such as feldspar and olivine [37]. In general, carbonation and hydrolysis are the most common geochemical mechanism on the Earth’s surface.

In hydrolysis reaction, ${\mathrm{Na}}^{+}$ and $K^{+}$ are mainly from feldspar, while ${\mathrm{Ca}}^{2+}$ and ${\mathrm{Mg}}^{2+}$ are derived from Ca-plagioclase and olivine [46]. After deducting atmospheric and anthropogenic inputs, remaining ${\mathrm{Na}}^{+}$ and $K^{+}$ ($K_{\mathrm{sil}}^{+}$ and ${\mathrm{Na}}_{\mathrm{sil}}^{+}$) are considered mainly from chemical weathering of silicate. Ideally, calcium and magnesium ions from silicate rocks (${\mathrm{Ca}}_{\mathrm{sil}}^{2+}$ and ${\mathrm{Mg}}_{\mathrm{sil}}^{2+}$) should be estimated in a monolithologic stream within the JRB [1]. However, the complex geological background hinders accurate measurements, so average (Ca/Na)_silicate bedrock_ = 0.43 ± 0.28 and (Mg/Na)_silicate bedrock_ = 0.26 ± 0.20 complied from some literatures are used in this study [47,48].

(2) $${\mathrm{Ca}}_{\mathrm{sil}}^{2+}=({\mathrm{Ca}}^{2+}{/\mathrm{Na}}^{+}){}_{\mathrm{silicate}\;\mathrm{bedrock}}\times {\mathrm{Na}}_{\mathrm{sil}}^{+}$$

(3) $${\mathrm{Mg}}_{\mathrm{sil}}^{2+}=({\mathrm{Mg}}^{2+}{/\mathrm{Na}}^{+}){}_{\mathrm{silicate}\;\mathrm{bedrock}}\times {\mathrm{Na}}_{\mathrm{sil}}^{+}$$

**Figure 5:**
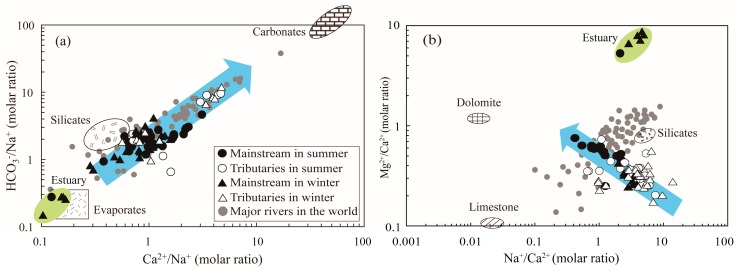
(**a**) Relationship between ${\mathrm{HCO}}_{3}^{-}$/${\mathrm{Na}}^{+}$ and ${\mathrm{Ca}}^{2+}$/${\mathrm{Na}}^{+}$ (molar ratios), (**b**) Plots of ${\mathrm{Na}}^{+}$/${\mathrm{Ca}}^{2+}$ and ${\mathrm{Mg}}^{2+}$/${\mathrm{Ca}}^{2+}$ (molar ratios) for river waters in the JR. Gray dots are the major rivers in the world [16]. End members in (**a**) are from Gaillardet, Dupre, Louvat and Allegre [16], and those of (**b**) are from Han and Liu [32].

Results of the above calculations show that the dissolved Ca and Mg from silicate weathering account for about 43.2% (5.4%–100%) for the total inputs from rock weathering. Although carbonate rocks cover a small area in the JRB, their contribution to solubility cannot be ignored owing to their high weathering rate of carbonate. For example, water samples from the JR were plotted as Na normalized molar ratios (Figure 5a), the distinct feature of dominant silicate weathering is observed. However, comparing with mainstream, some samples from tributaries covered by carbonate displays a stronger mixing signs between carbonate and silicate weathering. It is noticeable that some water samples from the estuary are located in evaporates endmember in Figure 5a. There is no obvious evaporates distributed in the study area, the reason for this phenomenon may be the influence of seawater that have low value of ${\mathrm{Ca}}^{2+}{/\mathrm{Na}}^{+}$ and ${\mathrm{HCO}}_{3}^{-}{/\mathrm{Na}}^{+}$ [43]. Figure 5b illustrates the relationship between ${\mathrm{Na}}^{+}$/${\mathrm{Ca}}^{2+}$ and ${\mathrm{Mg}}^{2+}$/${\mathrm{Ca}}^{2+}$ molar ratios for the JR waters. Compared with the major rivers in the world, the ${\mathrm{Mg}}^{2+}{/\mathrm{Ca}}^{2+}$ molar ratios are lower (0.2–5.8) in the river waters. Unlike the mixing trend showed by major rivers of the world between the silicates and limestone endmembers, the cations of the JR are potential affected by dolomite weathering. After atmospheric and anthropogenic and silicate weathering inputs are subtracted from concentrations in river waters, redundant ${\mathrm{Ca}}^{2+}$ and ${\mathrm{Mg}}^{2+}$ (${\mathrm{Ca}}_{\mathrm{carb}}^{2+}$ and ${\mathrm{Mg}}_{\mathrm{carb}}^{2+}$) are provided via carbonate weathering.

### 4.4. Chemical Budget

**Figure 6:**
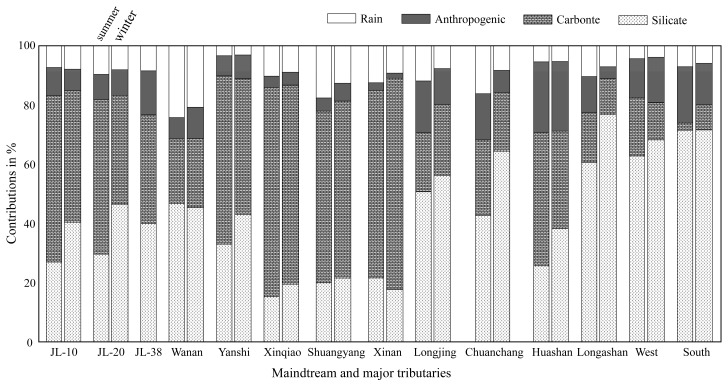
The contributions of different reservoirs (rain, anthropogenic inputs, silicate and carbonate weathering inputs) to the total cations (in mg/L). JL-10, JL-20 and JL-38 represent the upper, middle and lower reaches of mainstream, respectively.

Considering four major reservoirs (rain, anthropogenic inputs, silicate and carbonate weathering), we use an improved forward model based on mass budget equations to estimate relative contribution of different reservoirs to the total cations ($K^{+}$, ${\mathrm{Na}}^{+}$, ${\mathrm{Ca}}^{2+}$ and ${\mathrm{Mg}}^{2+}$) (act as mg/L) [1,32,49]. On the basis of the above discussion, for major ions X ([X]_river_) in the JR, the equation can be expressed as:

[X]_river_ = [X]_atm_ + [X]_anth_ + [X]_sil_ + [X]_carb_ + [X]_sulf_ (4)

where the suffix ‘atm’, ‘anth’, ‘sil’, ‘carb’ and ‘sulf’ represent atmospheric, anthropogenic, silicate weathering, carbonate weathering inputs and sulfide mineral, respectively. Because different ions can come from different reservoirs, simplifications in Equation (4) can be made. The contribution of different reservoirs to the cation load can be calculated in the mainstream (JL-10, JL-20 and JL-38) and major tributaries, and the result is illustrated in Figure 6. For all water samples, the dissolved cation load is controlled by atmospheric inputs (0.1%–70.8%), anthropogenic inputs (0–43.3%), silicate weathering inputs (13.0%–92.0%) and carbonate weathering inputs (0–70.3%). Overall, the cations of the JR are dominated by rocks weathering (carbonate and silicate weathering), which accounts for about 73.8% (summer) and 80.5% (winter) of the total cationic load for the mainstream. For tributaries, the contributions of silicate and carbonate weathering are highly variable owing to variation of rock types. The highest value is observed in the Longshan River and Xinqiao river, respectively. For densely populated areas, the cation contribution of human activities is large, especially in plains near estuary. Liu, Xu, Sun, Zhao, Shi and Liu [27] compiled the data of major ions in the JR in 2010, and mass balance calculations showed that anthropogenic inputs account for 14% (Punan) of the cations. In this study, the contribution of human activities (anthropogenic inputs) to the cations is lower (6.3%), indicating that the water environment has been effectively treated.

### 4.5. Consumption Flux of Atmospheric CO_2_ and Chemical Weathering

The material flux (in ton/a) derived from rock weathering can be estimated using the mass budget balance, as discussed in Section 4.4, and total water discharge as well as area of the JRB. Because of the seasonal changes in discharge, we assumed that discharge in summer is about 75% of the annual total discharge in the JR. In this study, the total cationic flux originated from silicate and carbonate weathering is estimated at the lower reaches. The calculated flux of silicate and carbonate weathering of the JRB are 3.7 × 10^5^ ton/a and 2.9 × 10^5^ ton/a, respectively. For the silicate weathering rate (SWR), it can be estimated using the cation and dissolved silica components supplied from silicate weathering, expressed in ton/km^2^/a.

(5) $$\mathrm{SWR}=({\mathrm{Ca}}_{\mathrm{sil}}^{2+}+{\mathrm{Mg}}_{\mathrm{sil}}^{2+}+K_{\mathrm{sil}}^{+}+{\mathrm{Na}}_{\mathrm{sil}}^{+}+{\mathrm{SiO}}_{2})\times \mathrm{discharge}/\mathrm{area}$$

Three samples (JL-10, JL-20 and JL-38) collected from the mainstream are used to calculate the rate of rock weathering corresponding to the upper reach (Zhangping), the middle reach (Punan) and the whole JRB (Longhai), respectively. In addition, dissolved silica in rivers might be absorbed by diatoms [42], thus the cationic weathering rate (abbreviated as ${\mathrm{Cat}}_{\mathrm{sil}}$) for silicates is also estimated reducing the error (Table 2). The silicate weathering in the JRB is characterized by ratios of ${\mathrm{Cat}}_{\mathrm{sil}}$ to SWR that are 0.2 to 0.8.

**Figure 7:**
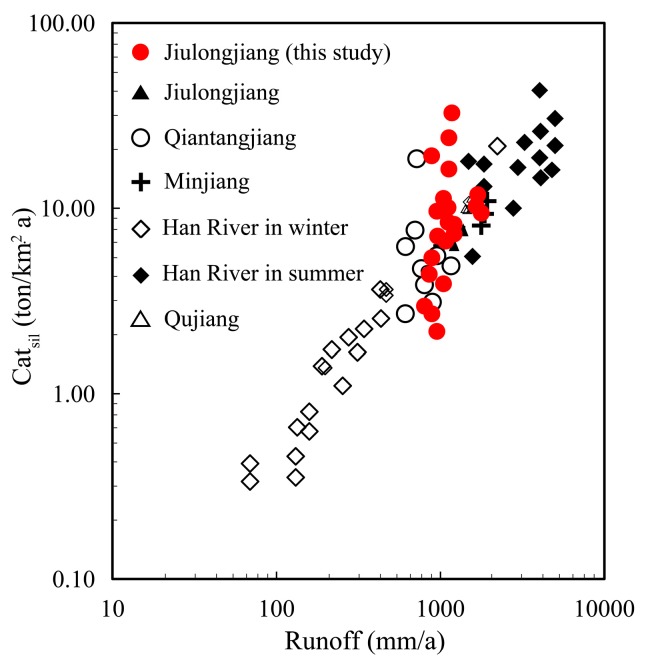
Relationship between Cat_sil_ (silicate weathering rate based on cations) and runoff (discharge/area). Data from the Han River [51], Qiantangjiang [52] and southeast coastal rivers of China (Jiulongjiang River, Minjiang and Qujiang River) [27].

There is a large difference of SWR in main tributaries regarding from 10.4 to 49.6 ton/km^2^/a. The highest SWR is observed in West river (49.6 ton/km^2^/a), which covered by granite relief with high discharge. For the main channel, the SWR increase slightly from upstream (Zhangping, 19.9 ton/km^2^/a) to downstream (Punan, 22.3 ton/km^2^/a), while there is a sharp increase in Longhai (53.2 ton/km^2^/a) regarded as a monitor for the entire basin. This sharp increase may be attributed to the confluence of the West river flowing through a silicate region. The total weathering rate of silicate (53.2 ton/km^2^/a) is 2.2 times that of the average silicate weathering rate (23.7 ton/km^2^/a) in the southeast coastal river basin [27]. The chemical weathering requires moisture. When other weathering conditions are the same, humid environments (high temperature and discharge) are often associated with high rates of rock weathering. For instance, there is a good linear relationship between the Cat_sil_ and runoff (discharge/area) in eastern Asia (Figure 7), and runoff regimes may be an important controlling factor for silicate weathering. High relief (mountainous area) can produce more steep slopes with rapid physical erosion rates. At the same time, a lot of fresh rocks are exposed to silicate chemical weathering [8]. However, high runoff and low residence times may impede continuous chemical weathering [50], for example, the Wanan River originated from the northwestern mountains and has a relatively low silicate weathering rate.

The ${\mathrm{CO}}_{2}$ uptake via silicate weathering (CSW) is calculated under the support of charge balance in silicate weathering reactions, and the unit of cations is mol/L.

(6) $$[{\mathrm{CO}}_{2}{]}_{\mathrm{SW}}=[{\mathrm{HCO}}_{3}{]}_{\mathrm{SW}}={(2\mathrm{Ca}}_{\mathrm{sil}}^{2+}+2{\mathrm{Mg}}_{\mathrm{sil}}^{2+}+K_{\mathrm{sil}}^{+}+{\mathrm{Na}}_{\mathrm{sil}}^{+})\times \mathrm{discharge}/\mathrm{area}$$

According to the above calculation, for the tributaries, the value of CSW ranges from 0.9 × 10^5^ mol/km^2^/a for Shuangyang river to 11.6 × 10^5^ mol/km^2^/a for Yanshi river, which causes carbon sink flux of 0.06 × 10^9^ mol/a to 3.8 × 10^9^ mol/a. For the whole basin, the rate of silicate weathering is 4.5 × 10^5^ mol/km^2^/a (6.4 × 10^9^ mol/a), this value is 4.5 times the global average of about 1 × 10^5^ mol/km^2^/a [16]. We assumed that the rate of silicate weathering is consistent on land surface, despite the great uncertainty, the ${\mathrm{CO}}_{2}$ sink by silicate weathering is about 0.8 Gt C/a. Compared to the ocean ${\mathrm{CO}}_{2}$ sink (2.4 ± 0.5 Gt C/a) and terrestrial biosphere ${\mathrm{CO}}_{2}$ sink (3.0 ± 0.8 Gt C/a) [7], the ${\mathrm{CO}}_{2}$ sink (0.8 Gt C/a) by silicate weathering is small, but it may be an important part in a budget imbalance (residual land sink) about 2.5 ± 1.3 Gt C/a [53].

In the weathering of carbonate, only one half of the bicarbonate ion is provided by calcite and the rest comes from the atmosphere, so the carbonate weathering rates (CWR) is estimated by using the ions derived from carbonate weathering.

(7) $$\mathrm{CWR}=({\mathrm{Ca}}_{\mathrm{carb}}^{2+}+{\mathrm{Mg}}_{\mathrm{carb}}^{2+}+0.5{\mathrm{HCO}}_{3}^{-})\times \mathrm{discharge}/\mathrm{area}$$

CWR represents approximately 6.3 to 48.4 ton/km^2^/a in main tributaries. The highest CWR is observed in Yanshi river (48.4 ton/km^2^/a). Additionally, the weathering rate of carbonate decrease significantly from upper to lower JRB. This characteristic may be attributed to the rock type (carbonate in the upper JRB, silicate elsewhere) displayed in Figure 1. However, despite the lack of large area carbonate outcrops in some tributaries, some streams with siliceous bedrocks have relatively high rates of weathering of carbonates such as Huashan River and West River. That is probably because trace calcite minerals in silicates may contribute a significant amount of calcium and magnesium [43]. ${\mathrm{CO}}_{2}$ consumption in the carbonate weathering process ranges from 0.3 × 10^5^ mol/km^2^/a for West river to 10.4 × 10^5^ mol/km^2^/a for Yanshi river. The total rock weathering rates (TWR) for the JRB represents about 68.2 ton/km^2^/a (Longhai, JL-38), and it is higher than the estimated average value (24 ton/km^2^/a) of major rivers in the world [16]. Perhaps the higher runoff (1200 mm/a) and appropriate temperature (19.9 °C to 21.1 °C) are a reasonable explanation put forward by many scholars [8].

### 4.6. Sulfuric and Nitric Acid As Weathering Agent

Except for $H_{2}{\mathrm{CO}}_{3}$, other inorganic acids ($H_{2}{\mathrm{SO}}_{4}$ and ${\mathrm{HNO}}_{3}$) contribute to the dissolution of rocks [54]. For example, in the major rivers of the South Korean, the actual atmospheric CO_2_ consumption will be reduced to 64.9% of the value estimated when carbonic acid provides all the protons in the weathering reactions. If above acids provide the protons that are necessary for the weathering reaction, carbonate weathering processes may lead to a net carbon dioxide emission in a short time. In geological time scales (more than 1 Ma), silicate weathering may lose ability to regulate the ${\mathrm{CO}}_{2}$ level in the atmosphere when sulfuric acid and nitric acid are involved in the weathering process [22,23,54,55,56,57].

**Figure 8:**
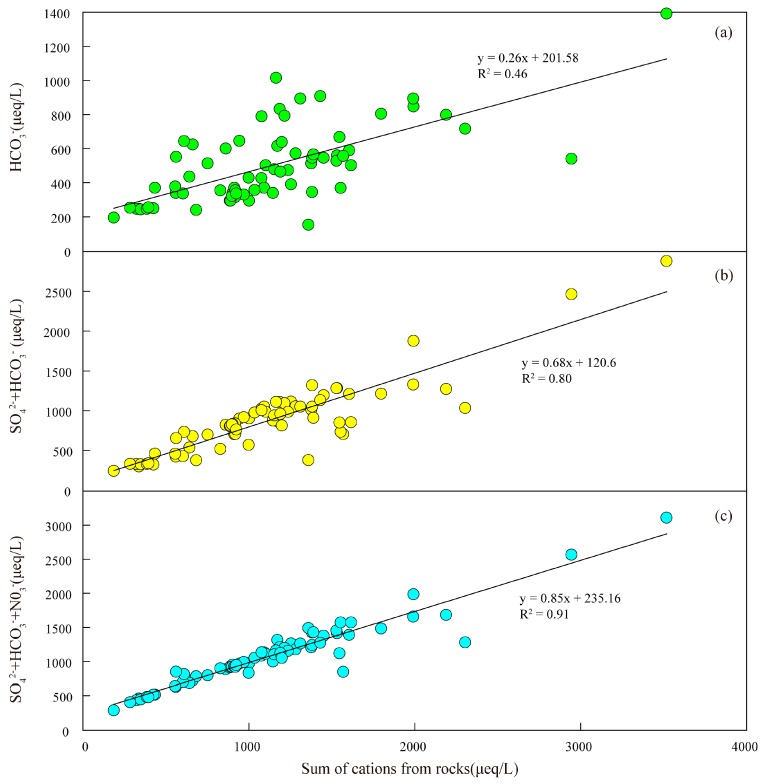
Relationship between the sum of the cations from rocks (μeq/L) weathering and ${\mathrm{HCO}}_{3}^{-}$ (**a**), and ${\mathrm{HCO}}_{3}^{-}$ + ${\mathrm{SO}}_{4}^{2-}$ (**b**), and ${\mathrm{HCO}}_{3}^{-}$ + ${\mathrm{SO}}_{4}^{2-}$ + ${\mathrm{NO}}_{3}^{-}$ (**c**) in the JR.

In the JR waters, sum of cations from rocks could not be balanced by ${\mathrm{HCO}}_{3}^{-}$ alone (${\mathrm{HCO}}_{3}^{-}$/(sum of cations from rocks) <0.5) (Figure 8a), this implies that there are other anions that participate in the ion equilibrium. In the basin system, sulfuric acid usually comes from the oxidation of sulfide minerals and acid rain. In Section 4.1, we estimated that about half of ${\mathrm{SO}}_{4}^{2-}$ in the river is supplied by acid rain. Although the contribution of the rain to ${\mathrm{NO}}_{3}^{-}$ is small (about 32%) comparing to the contribution of the agricultural wastewater, only ${\mathrm{HCO}}_{3}^{-}$ and ${\mathrm{SO}}_{4}^{2-}$ could not be balanced by sum of cations from rocks (Figure 8b), while the sum of cations from rocks are almost balanced by ${\mathrm{HCO}}_{3}^{-}$, ${\mathrm{SO}}_{4}^{2-}$ and ${\mathrm{NO}}_{3}^{-}$. This indicated that the effect of nitric acid and sulfuric acid on weathering should be considered simultaneously in the study area.

According the estimation method reported by Moon, Huh, Qin and van Pho [1], ${\mathrm{CO}}_{2}$ consumption rate can be calculated under the effects of acid rain in the silicate and carbonate weathering process, as follows:

(8) $$[{\mathrm{CO}}_{2}{]}_{\mathrm{SSW}}=[{\mathrm{CO}}_{2}{]}_{\mathrm{SW}}-\gamma \times (2[{\mathrm{SO}}_{4}^{2-}]+[{\mathrm{NO}}_{3}^{-}]){}_{\mathrm{rain}}\times \mathrm{discharge}/\mathrm{area}$$

(9) $$[{\mathrm{CO}}_{2}{]}_{\mathrm{SCW}}=[{\mathrm{CO}}_{2}{]}_{\mathrm{CW}}-(1-\gamma )\times (2[{\mathrm{SO}}_{4}^{2-}]+[{\mathrm{NO}}_{3}^{-}]{)}_{\mathrm{rain}}\times \mathrm{discharge}/\mathrm{area}$$

where γ is a variable factor (γ = Cat_sil_/(Cat_sil_ + Cat_carb_)). In the major tributaries, ${\mathrm{CO}}_{2}$ consumption rates by silicates and carbonates are from 0.5 × 10^5^ mol/km^2^/a to 10.8 × 10^5^ mol/km^2^/a and from 0.3 × 10^5^ mol/km^2^/a to 10.1 × 10^5^ mol/km^2^/a when sulfuric acid and nitric acid are considered in the weathering process, and the ratios of SSW/CSW and SCW/CCW are between 0.4 and 0.9 (mean 0.76) and between 0.8 and 1 (mean 0.90). These results suggest that the silicate weathering reaction is more easily accelerated by strong acid in precipitation. For the whole basin, the actual ${\mathrm{CO}}_{2}$ consumption via carbonate weathering (SCW) and silicate weathering (SSW) are 2.2 × 10^5^ mol/km^2^/a and 3.7 × 10^5^ mol/km^2^/a, respectively. The actual rate of absorption of carbon dioxide in the JRB is 85.5% (or less) of the value without considering nitric acid and sulfuric acid. Consequently, the carbon sink may be overestimated about 1.5 × 10^9^ mol/a (17.4 Gg C/a), and this value is about 27.0% of carbon uptake via silicate weathering estimated in the JRB. These results show that the amount of ${\mathrm{CO}}_{2}$ consumption in the atmosphere by carbonate and silicate weathering, will be overestimated when the effects of $H_{2}{\mathrm{SO}}_{4}$ and ${\mathrm{HNO}}_{3}$ are ignored in the process of calculating the carbon sink.

## 5. Conclusions

We have investigated the dissolved major ions concentrations in the JRB, where silicate rocks occupy the middle and lower reaches while carbonate rocks are mainly concentrated in the upper reaches. In the water samples, the average TDS values of the JR is 99.2 mg/L in summer and 133.9 mg/L in winter with an annual average of 116.6 mg/L. The river water is characterized by slight alkalinity and medium concentrations of ${\mathrm{Ca}}^{2+}$ and ${\mathrm{HCO}}_{3}^{-}$, and both ions constitute approximately 45.4% of the total ionic budget. Except ${\mathrm{HCO}}_{3}^{-}$, the second common anion is ${\mathrm{SO}}_{4}^{2-}$ (1.4 to 92.3 mg/L), and about half of ${\mathrm{SO}}_{4}^{2-}$ came from the rainfall (55.4% in summer and 49.5% in winter). A mass balance calculation indicated that the cations of the river waters are mainly from rocks (silicates and carbonates) accounting for 74.0% (39.5%–100%) of the total cationic loads. Other sources such as atmospheric deposition and anthropogenic inputs account for 15.6% (0.1%–70.8%) and 10.4% (0–43.3%), respectively. The JR, which drains the densely populated areas, which is serious influenced by human activities, and highest contribution of anthropogenic inputs for cations up to 43.3%.

The JRB has higher chemical weathering rates (SWR, 53.2 ${\mathrm{ton}/\mathrm{km}}^{2}/a$ and CWR, 15.0 ${\mathrm{ton}/\mathrm{km}}^{2}/a$). In addition, this study also shows that chemical weathering rates in the JRB is controlled by rock type and runoff. The actual rate of absorption of carbon dioxide in the JRB is 85.5% (or less) of the value without considering nitric acid and sulfuric acid. So ${\mathrm{CO}}_{2}$ consumption may be overestimated by 1.5 × 10^9^ mol/a (17.4 Gg C/a), and this value is about 27.0% of the ${\mathrm{CO}}_{2}$ consumption via silicate weathering in the JRB. These results emphasize that human activities such as industry and agriculture can potentially influence chemical weathering process of rocks or minerals and the associated long-term carbon cycle.

## Figures and Tables

**Table 1:** Cl-normalized molar ratios in rainwater within the Jiulongjiang River basin (JRB).

City	Date	pH	SO_4_/Cl	NO_3_/Cl	Na/Cl	K/Cl	Ca/Cl	Mg/Cl
Xiamen ^a^	2002	4.6	1.3	0.9	1.5	0.2	0.9	0.2
Longyan ^a^	2002	5.9	0.6	0.1	0.6	0.2	0.9	0.1
Zhangzhou ^b^	2012	6.1	1.8	1.0	0.2	0.2	1.7	0.2
Zhangzhou ^b^	2011	6.2	1.8	0.9	1.3	0.3	1.5	0.4
Xiamen ^c^	2000–2015	4.7	1.0	0.8	0.8	0.1	0.8	0.2
Xiamen ^c^	2000–2015	4.7	1.4	1.4	0.9	0.3	0.9	0.2
Average		5.4	1.3	0.9	0.9	0.2	1.1	0.2

^a^ [38]; ^b^ [39]; ^c^ [40].

**Table 2:** Chemical weathering rates and associated CO_2_ consumption in the JRB.

River	Discharge 10^9^ m^3^/a	Area 10^3^ km^2^	Silicates		Carbonates		CO_2_ Consumption Rates			
			${\mathrm{Cat}}_{\mathrm{sil}}$ ^a^	SWR ^b^	CWR ^b^	TWR ^b^	CSW ^c^	CCW ^c^	SSW ^d^	SCW ^d^
			ton/km^2^/a		10^5^ mol/km^2^/a					
Mainstream										
Zhangping	5.5	4.9	9.3	19.9	26.4	46.3	4.5	4.8	3.8	4.4
Punan	10.2	8.5	10.5	22.3	22.3	44.6	4.4	3.7	2.8	2.6
Longhai	14.5	14.1	40.8	53.2	15.0	68.2	4.5	2.4	3.7	2.2
Tributaries										
Wanan	1.5	1.5	4.1	14.0	6.3	20.2	1.7	0.5	0.7	0.4
Yanshi	1.5	1.5	23.9	32.9	48.4	81.3	11.6	10.4	10.8	10.1
Xinqiao	0.8	1.0	2.8	11.7	24.3	36.0	1.2	3.5	0.9	3.0
Shuangyang	0.6	0.7	2.2	10.4	14.9	25.2	0.9	1.9	0.5	1.5
Xinan	0.5	0.7	2.8	11.9	21.5	33.4	1.2	2.6	0.9	2.4
Longjing	0.9	0.9	10.0	23.2	11.2	34.4	4.7	1.0	3.8	0.9
Chuanchang	1.2	1.0	9.3	23.3	10.2	33.5	4.3	1.0	3.2	0.9
Huashan	1.1	1.1	11.5	28.9	20.3	49.2	5.8	4.7	5.0	4.3
Longshan	1.3	1.2	16.8	33.5	14.9	48.4	8.0	0.9	6.6	0.8
West	4.3	3.9	34.1	49.6	28.7	78.3	5.9	3.5	5.0	3.2
South	0.6	0.7	19.7	35.9	9.6	45.5	9.4	0.3	8.3	0.3

^a^ Cat_sil_: the cation-silicate weathering rates. ^b^ SWR: silicate weathering rates; CWR: carbonate weathering rates; TWR: total rock weathering rates. ^c^ ${\mathrm{CO}}_{2}$ consumption rates (in mol/km^2^/a) are calculated when all the $H^{+}$ involved in the process are from $H_{2}{\mathrm{CO}}_{3}$. ^d^ Estimated ${\mathrm{CO}}_{2}$ consumption rates (in mol/km^2^/a) by silicate weathering and carbonate weathering when sulfuric acid and nitric acid are considered in the weathering process.

## Supplementary Materials

The following are available online at http://www.mdpi.com/1660-4601/16/3/440/s1, Table S1: The physical–chemical parameters and major ions concentration in the JR.

## References

[1] Moon S., Huh Y., Qin J., van Pho N. (2007). Chemical weathering in the Hong (Red) River basin: Rates of silicate weathering and their controlling factors. Geochim. Cosmochim. Acta.

[2] Caves J.K., Jost A.B., Lau K.V., Maher K. (2016). Cenozoic carbon cycle imbalances and a variable weathering feedback. Earth Planet. Sci. Lett..

[3] White A.F., Blum A.E., Bullen T.D., Vivit D.V., Schulz M., Fitzpatrick J. (1999). The effect of temperature on experimental and natural chemical weathering rates of granitoid rocks. Geochim. Cosmochim. Acta.

[4] Oliva P., Viers J., Dupré B. (2003). Chemical weathering in granitic environments. Chem. Geol..

[5] Suchet P.A., Probst J.-L., Ludwig W. (2003). Worldwide distribution of continental rock lithology: Implications for the atmospheric/soil CO_2_ uptake by continental weathering and alkalinity river transport to the oceans. Glob. Biogeochem. Cycles.

[6] Wu W., Xu S., Yang J., Yin H. (2008). Silicate weathering and CO_2_ consumption deduced from the seven Chinese rivers originating in the Qinghai-Tibet Plateau. Chem. Geol..

[7] Le Quéré C., Andrew R.M., Friedlingstein P., Sitch S., Pongratz J., Manning A.C., Korsbakken J.I., Peters G.P., Canadell J.G., Jackson R.B. (2018). Global Carbon Budget 2017. Earth Syst. Sci. Data.

[8] Goudie A.S., Viles H.A. (2012). Weathering and the global carbon cycle: Geomorphological perspectives. Earth-Sci. Rev..

[9] Berner R.A., Caldeira K. (1998). The need for mass balance and feedback in the geochemical carbon cycle. Geology.

[10] Kump L.R., Brantley S.L., Arthur M.A. (2000). Chemical Weathering, Atmospheric CO_2_, and Climate. Annu. Rev. Earth Planet. Sci..

[11] Li Y., Cao W., Su C., Hong H. (2011). Nutrient sources and composition of recent algal blooms and eutrophication in the northern Jiulong River, Southeast China. Mar. Pollut. Bull..

[12] Zhao Z., Liu G., Liu Q., Huang C., Li H. (2018). Studies on the Spatiotemporal Variability of River Water Quality and Its Relationships with Soil and Precipitation: A Case Study of the Mun River Basin in Thailand. Int. J. Environ. Res. Public Health.

[13] Wang L., Zhang L., Cai W.-J., Wang B., Yu Z. (2016). Consumption of atmospheric CO_2_ via chemical weathering in the Yellow River basin: The Qinghai–Tibet Plateau is the main contributor to the high dissolved inorganic carbon in the Yellow River. Chem. Geol..

[14] Xu Z., Liu C.-Q. (2010). Water geochemistry of the Xijiang basin rivers, South China: Chemical weathering and CO_2_ consumption. Appl. Geochem..

[15] Chetelat B., Liu C.Q., Zhao Z.Q., Wang Q.L., Li S.L., Li J., Wang B.L. (2008). Geochemistry of the dissolved load of the Changjiang Basin rivers: Anthropogenic impacts and chemical weathering. Geochim. Cosmochim. Acta.

[16] Gaillardet J., Dupre B., Louvat P., Allegre C.J. (1999). Global silicate weathering and CO_2_ consumption rates deduced from the chemistry of large rivers. Chem. Geol..

[17] Millot R., Gaillardet J., Dupré B., Allègre C.J. (2002). The global control of silicate weathering rates and the coupling with physical erosion: New insights from rivers of the Canadian Shield. Earth Planet. Sci. Lett..

[18] Tang Y., Han G., Li F., Wu Q. (2016). Natural and anthropogenic sources of atmospheric dust at a remote forest area in Guizhou Karst region, Southwest China. Geochem. Explor. Environ. Anal..

[19] Li X.-D., Liu C.-Q., Harue M., Li S.-L., Liu X.-L. (2010). The use of environmental isotopic (C, Sr, S) and hydrochemical tracers to characterize anthropogenic effects on karst groundwater quality: A case study of the Shuicheng Basin, SW China. Appl. Geochem..

[20] Wu Q., Han G. (2017). δ^13^C_DIC_ tracing of dissolved inorganic carbon sources at Three Gorges Reservoir, China. Water Sci. Technol..

[21] Liang B., Han G., Liu M., Yang K., Li X., Liu J. (2018). Distribution, Sources, and Water Quality Assessment of Dissolved Heavy Metals in the Jiulongjiang River Water, Southeast China. Int. J. Environ. Res. Public Health.

[22] Li S.-L., Calmels D., Han G., Gaillardet J., Liu C.-Q. (2008). Sulfuric acid as an agent of carbonate weathering constrained by δ13CDIC: Examples from Southwest China. Earth Planet. Sci. Lett..

[23] Shin W.-J., Ryu J.-S., Park Y., Lee K.-S. (2011). Chemical weathering and associated CO_2_ consumption in six major river basins, South Korea. Geomorphology.

[24] Lerman A., Wu L., Mackenzie F.T. (2007). CO_2_ and H_2_SO_4_ consumption in weathering and material transport to the ocean, and their role in the global carbon balance. Mar. Chem..

[25] Li X.-D., Liu C.-Q., Liu X.-L., Bao L.-R. (2011). Identification of dissolved sulfate sources and the role of sulfuric acid in carbonate weathering using dual-isotopic data from the Jialing River, Southwest China. J. Asian Earth Sci..

[26] Amiotte Suchet P., Probst A., Probst J.L. (1995). Influence of acid rain on CO_2_ consumption by rock weathering: Local and global scales. Water Air Soil Pollut..

[27] Liu W., Xu Z., Sun H., Zhao T., Shi C., Liu T. (2018). Geochemistry of the dissolved loads during high-flow season of rivers in the southeastern coastal region of China: Anthropogenic impact on chemical weathering and carbon sequestration. Biogeosciences.

[28] Huang J., Zhang Z., Feng Y., Hong H. (2013). Hydrologic response to climate change and human activities in a subtropical coastal watershed of southeast China. Reg. Environ. Chang..

[29] Yang K., Han G., Liu M., Li X., Liu J., Zhang Q. (2018). Spatial and Seasonal Variation of O and H Isotopes in the Jiulong River, Southeast China. Water.

[30] Cao W., Hong H., Yue S. (2005). Modelling agricultural nitrogen contributions to the Jiulong River estuary and coastal water. Glob. Planet. Chang..

[31] Meybeck M. (2003). Global Occurrence of Major Elements in Rivers. Treatise Geochem..

[32] Han G., Liu C.-Q. (2004). Water geochemistry controlled by carbonate dissolution: A study of the river waters draining karst-dominated terrain, Guizhou Province, China. Chem. Geol..

[33] Depetris P.J., Pasquini A.I., Lecomte K.L. (2014). Weathering and the Riverine Denudation of Continents.

[34] Rivé K., Gaillardet J., Agrinier P., Rad S. (2013). Carbon isotopes in the rivers from the Lesser Antilles: Origin of the carbonic acid consumed by weathering reactions in the Lesser Antilles. Earth Surf. Process. Landf..

[35] Rao W., Han G., Tan H., Jin K., Wang S., Chen T. (2017). Chemical and Sr isotopic characteristics of rainwater on the Alxa Desert Plateau, North China: Implication for air quality and ion sources. Atmos. Res..

[36] Martin J.M., Meybeck M. (1979). Elemental mass-balance of material carried by major world rivers. Mar. Chem..

[37] Moosdorf N., Hartmann J., Lauerwald R., Hagedorn B., Kempe S. (2011). Atmospheric CO_2_ consumption by chemical weathering in North America. Geochim. Cosmochim. Acta.

[38] Lin W.-S. (2014). Analysis on the Characteristics of Chemical Composition of Precipitation in Zhangzhou City, Fujian Province. Guangzhou Chem. Ind..

[39] Zhao W.-H. (2004). An Analysis on the Changing Trend of Acid Rain and its Causes in Fujian Province. Fujian Geogr..

[40] Eanet (2016). EANET Data on the Acid Deposition in the East Asian Region. http://www.eanet.asia.

[41] Yu J., Wang J., Zhao L., Zhang Y. (2014). Study on the Source of Sulfure and Nitrogen in Acid Rain bu Using Sulfure and Nitrogen Stable Isotopic Technology. J. Henan Norm. Univ. (Nat. Sci. Ed.).

[42] Roy S., Gaillardet J., Allegre C.J. (1999). Geochemistry of dissolved and suspended loads of the Seine River, France: Anthropogenic impact, carbonate and silicate weathering. Geochim. Cosmochim. Acta.

[43] Hagedorn B., Cartwright I. (2009). Climatic and lithologic controls on the temporal and spatial variability of CO_2_ consumption via chemical weathering: An example from the Australian Victorian Alps. Chem. Geol..

[44] Wu Q., Han G. (2015). Sulfur isotope and chemical composition of the rainwater at the Three Gorges Reservoir. Atmos. Res..

[45] Wu Y., Wang X., Li Y., Ya M., Luo H., Hong H. (2017). Polybrominated diphenyl ethers, organochlorine pesticides, and polycyclic aromatic hydrocarbons in water from the Jiulong River Estuary, China: Levels, distributions, influencing factors, and risk assessment. Environ. Sci. Pollut. Res. Int..

[46] Mortatti J., Probst J.-L. (2003). Silicate rock weathering and atmospheric/soil CO_2_ uptake in the Amazon basin estimated from river water geochemistry: Seasonal and spatial variations. Chem. Geol..

[47] Zhang C., Su H., Yu M., Hu Z. (2012). Zircon U-Pb age and Nd-Sr-Pb isotopic characteristics of Dayang-Juzhou granite in Longyan, Fujian Province and its geological significance. Acta Petrol. Sin..

[48] Wang S., Zhang D., Vatuva A., Yan P., Ma S., Feng H., Yu T., Bai Y., Di Y. (2015). Zircon U-Pb geochronology, geochemistry and Hf isotope compositions of the Dayang and Juzhou granites in Longyan, Fujian and their geological implication. Geochimica.

[49] Galy A., France-Lanord C. (1999). Weathering processes in the Ganges–Brahmaputra basin and the riverine alkalinity budget. Chem. Geol..

[50] Maher K. (2010). The dependence of chemical weathering rates on fluid residence time. Earth Planet. Sci. Lett..

[51] Ryu J.-S., Lee K.-S., Chang H.-W., Shin H.S. (2008). Chemical weathering of carbonates and silicates in the Han River basin, South Korea. Chem. Geol..

[52] Liu W., Jiang H., Shi C., Zhao T., Liang C., Hu J., Xu Z. (2016). Chemical and strontium isotopic characteristics of the rivers around the Badain Jaran Desert, northwest China: Implication of river solute origin and chemical weathering. Environ. Earth Sci..

[53] Ciais P., Sabine C., Bala G., Bopp L., Brovkin V., Canadell J., Chhabra A., DeFries R., Galloway J., Heimann M., Stocker T.F., Qin D., Plattner G.-K., Tignor M., Allen S.K., Boschung J., Nauels A., Xia Y., Bex V., Midgley P.M. (2014). Carbon and Other Biogeochemical Cycles. Climate Change 2013: The Physical Science Basis. Contribution of Working Group I to the Fifth Assessment Report of the Intergovernmental Panel on Climate Change.

[54] Lerman A., Wu L. (2006). CO_2_ and sulfuric acid controls of weathering and river water composition. J. Geochem. Explor..

[55] Yuan F., Mayer B. (2012). Chemical and isotopic evaluation of sulfur sources and cycling in the Pecos River, New Mexico, USA. Chem. Geol..

[56] Xu Z., Liu C.-Q. (2007). Chemical weathering in the upper reaches of Xijiang River draining the Yunnan–Guizhou Plateau, Southwest China. Chem. Geol..

[57] Spence J., Telmer K. (2005). The role of sulfur in chemical weathering and atmospheric CO_2_ fluxes: Evidence from major ions, δ^13^C_DIC_, and δ^34^S_SO4_ in rivers of the Canadian Cordillera. Geochim. Cosmochim. Acta.

