# Supplementary material for: Hydro-Geochemistry of the River Water in the Jiulongjiang River Basin, Southeast China: Implications of Anthropogenic Inputs and Chemical Weathering
Source: Int J Environ Res Public Health. 2019 Feb 2;16(3):440. doi: 10.3390/ijerph16030440 (PMC6388126; doi:10.3390/ijerph16030440)
Supplement: Supplementary file 1 [file ijerph-16-00440-s001.pdf]

Table S1. The physical-chemical parameters and major ions concentration in the JR.

Samples	Rivers	T	EC	pH	Na^+	K^+	Ca^{2+}	Mg^{2+}	Cl^-	SO_4^{2-}	NO_3^-	HCO_3^-	SiO_2	TDS[a]	TZ^+	TZ^-	NICB[b]
		(°C)	(μs/cm)		(mg/L)										(ueq/L)		(%)
Summer																	
JL-1	Wanan R.	21.1	17.8	7.6	1.6	1.1	1.5	0.3	0.9	1.4	0.9	7.5	11.8	23.2	194.2	192.9	0.7
JL-2	Wanan R.	23.6	27.6	7.0	2.0	1.4	2.1	0.4	1.0	2.9	2.4	7.9	12.5	28.6	265.6	255.4	3.8
JL-3	Wanan R.	23.4	41.3	6.7	2.1	1.8	3.5	0.9	1.2	5.4	3.9	11.5	12.7	37.2	386.6	398.1	-3.0
JL-4	Yanshi R.	25.0	359.8	7.0	13.6	2.5	29.9	13.2	23.6	92.3	6.2	32.9	11.3	209.1	3233.7	3228.9	0.2
JL-5	North R.	24.1	162.6	7.0	2.9	2.1	15.7	7.2	2.8	46.7	6.5	21.1	12.6	106.9	1552.4	1500.8	3.3
JL-6	Xinqiao R.	25.0	102.1	7.7	1.8	1.7	13.2	2.8	2.0	10.6	4.1	36.6	13.8	68.3	1014.5	942.5	7.1
JL-7	Shuangyang R.	23.6	57.6	7.3	1.5	1.4	7.1	1.5	1.2	4.3	3.5	22.5	11.6	43.3	580.0	548.8	5.4
JL-8	Xinqiao R.	25.5	111.9	7.9	1.9	1.8	14.4	3.1	2.1	12.1	4.3	39.4	14.2	73.6	1098.5	1027.0	6.5
JL-9	North R.	24.6	151.7	7.1	2.6	1.9	13.1	5.0	2.2	28.7	3.7	26.0	12.5	82.6	1228.9	1144.1	6.9
JL-10	North R.	25.5	162.5	7.1	5.5	2.0	14.6	5.5	7.6	34.6	9.4	23.8	16.9	107.8	1469.3	1475.1	-0.4
JL-11	Xinan R.	24.8	81.1	7.5	2.0	1.6	10.9	1.7	1.6	2.6	2.9	38.1	14.7	56.9	806.4	769.1	4.6
JL-12	North R.	24.6	134.4	7.1	3.6	2.1	12.9	4.9	2.9	32.7	5.0	22.5	12.8	88.0	1256.3	1210.4	3.7
JL-13	Xiazhe R.	25.3	35.3	7.4	2.3	2.1	2.7	0.3	1.3	2.2	2.8	11.9	18.0	37.6	313.7	322.6	-2.9
JL-14	North R.	24.5	127.3	6.9	3.5	2.2	12.0	4.4	4.5	29.1	4.4	17.9	12.2	81.2	1170.7	1097.7	6.2
JL-15	North R.	25.2	130.0	7.3	3.3	2.1	12.6	4.5	2.7	29.6	4.8	21.8	13.6	84.0	1196.6	1127.8	5.8
JL-16	Xiandu R.	24.9	55.3	7.6	3.6	2.3	4.5	1.0	1.7	2.5	10.0	15.0	19.2	52.2	521.8	506.2	3.0
JL-17	North R.	25.0	125.3	7.2	3.3	2.1	11.8	4.2	3.2	28.1	4.7	20.1	13.2	80.6	1133.4	1079.6	4.8
JL-18	North R.	25.5	123.1	7.2	4.0	2.1	11.0	3.8	4.5	25.1	5.1	19.2	11.9	77.1	1090.5	1046.7	4.0
JL-19	North R.	25.8	122.0	7.2	4.0	2.1	10.8	3.7	4.5	24.4	6.8	17.9	12.3	77.6	1069.4	1040.0	2.8
JL-20	North R.	25.9	123.5	7.1	4.1	2.1	10.9	3.6	4.7	24.7	7.2	17.9	12.4	78.7	1075.7	1057.3	1.7
JL-21	Longjing R.	31.9	96.2	7.6	5.3	3.4	6.8	1.4	4.3	4.0	12.9	20.7	18.5	66.9	775.4	749.0	3.4
JL-22	North R.	26.4	128.1	7.0	4.1	2.4	11.3	3.5	3.9	23.6	7.4	20.7	13.0	79.5	1085.7	1061.0	2.3
JL-23	North R.	28.4	130.6	7.1	4.0	2.4	10.9	3.6	4.1	24.2	6.0	19.6	13.4	78.4	1074.4	1038.2	3.4
JL-24	Chuanchang R.	24.3	66.0	7.2	2.5	2.7	5.0	0.8	1.7	3.9	6.8	15.0	13.6	44.4	490.8	484.8	1.2
JL-25	Chuanchang R.	25.1	59.0	7.3	3.3	2.8	4.9	0.8	2.1	3.9	7.4	14.8	15.7	48.3	521.4	503.6	3.4
JL-26	Chuanchang R.	25.0	78.0	7.4	3.8	2.9	5.2	0.9	2.0	3.9	8.8	15.6	16.2	51.5	575.0	534.8	7.0
JL-27	Chuanchang R.	24.9	74.0	7.4	3.2	2.7	5.5	1.0	2.3	4.0	9.4	15.0	16.4	52.0	569.9	545.3	4.3
JL-28	Huashan R.	28.0	198.0	6.7	6.6	7.9	17.2	4.6	8.2	10.7	69.0	9.3	21.4	150.4	1730.7	1721.0	0.6
JL-29	West R.	25.5	118.0	7.1	4.3	4.3	9.4	2.1	4.3	6.5	25.2	14.6	20.3	83.6	939.0	902.3	3.9
JL-30	Longshan R.	26.4	91.0	6.9	6.5	2.9	7.5	1.2	4.5	4.8	9.3	26.5	19.3	69.1	826.8	809.4	2.1
JL-31	West R.	27.3	124.0	7.0	6.5	4.9	10.3	2.3	5.9	7.8	23.6	21.6	22.4	94.4	1109.7	1062.2	4.3
JL-32	West R.	28.0	180.0	6.9	9.1	5.8	14.9	2.6	10.7	11.9	28.0	37.5	18.8	120.7	1504.1	1617.1	-7.5
JL-33	West R.	27.8	236.0	7.0	17.2	7.0	18.3	3.5	21.1	19.6	16.7	49.0	18.7	146.7	2130.5	2078.0	2.5
JL-34	South R.	28.8	77.4	7.3	4.6	3.5	5.0	1.1	3.6	3.5	12.0	15.2	21.5	62.3	631.8	617.0	2.4
JL-35	South R.	29.6	105.8	7.0	6.2	4.0	7.0	1.6	5.2	4.3	17.0	20.5	24.8	80.3	847.6	846.9	0.1
JL-36	South R.	31.5	172.0	7.0	13.0	5.1	8.0	2.6	16.9	6.7	16.3	26.2	24.5	106.1	1305.2	1307.6	-0.2
JL-37	South R.	31.0	572.0	7.0	64.7	7.9	13.1	9.9	110.4	20.7	12.8	38.4	20.4	279.0	4483.0	4380.2	2.3
JL-38	Estuary	27.3	37.9	7.0	6.1	3.6	11.1	2.9	6.7	15.9	13.9	22.5	16.2	87.6	1148.5	1114.4	3.0
JL-39	Estuary	27.2	139.7	7.0	6.3	3.5	10.9	3.0	7.4	16.9	13.2	21.6	15.5	87.5	1154.0	1127.1	2.3
JL-40	Estuary	27.1	135.1	7.0	5.9	3.0	10.9	3.3	6.8	20.4	11.4	20.5	14.6	86.5	1145.6	1136.6	0.8
JL-41	Estuary	27.9	1482.0	7.0	189.1	11.2	19.5	26.4	367.2	6.8	50.3	32.0	14.2	700.7	11650.0	11836.0	-1.6
JL-42	Estuary	27.0	160.6	7.1	8.1	3.2	12.3	3.9	10.8	25.7	7.7	20.7	12.0	94.0	1369.1	1303.2	4.8

Continued on next page

Samples	Rivers	T	EC	pH	Na^+	K^+	Ca^{2+}	Mg^{2+}	Cl^-	SO_4^{2-}	NO_3^-	HCO_3^-	SiO_2	TDS[a]	TZ^+	TZ^-	NICB[b]
		(°C)	(μs/cm)		(mg/L)										(ueq/L)		(%)
Winter																	
JL-1	Wanan R.	11.0	26.8	7.0	1.6	1.1	1.9	0.3	1.1	2.0	1.8	7.6	10.2	23.8	215.2	227.7	-5.8
JL-2	Wanan R	15.2	33.5	6.3	2.2	1.7	2.7	0.6	1.2	3.7	3.0	12.4	11.3	32.7	327.6	361.6	-10.4
JL-3	Wanan R	15.5	66.6	6.9	3.0	2.7	5.1	1.1	1.5	7.8	7.0	15.9	11.3	47.4	540.6	579.1	-7.1
JL-4	Yanshi R	14.6	430	7.2	21.4	5.6	43.6	8.0	28.5	71.5	14.1	84.9	11.2	246.3	3902.6	3912.2	-0.2
JL-5	North R	18.7	150.3	7.1	6.7	3.3	14.4	2.9	6.6	23.2	7.9	30.6	12.8	92.9	1327.6	1297.3	2.3
JL-6	Xinqiao R	12.5	137.9	7.4	2.9	2.8	18.5	3.1	2.5	13.3	6.2	50.8	10.3	84.9	1378.9	1278.2	7.3
JL-7	Shuangyang R	15.1	97.2	7.4	2.3	2.8	12.5	1.9	2.2	8.8	6.3	31.3	9.1	61.3	944.2	858.5	9.1
JL-8	Xinqiao R	14.5	138.9	7.8	2.9	2.8	19.2	3.1	2.6	14.4	6.6	48.3	8.8	84.5	1411.8	1270.6	10.0
JL-9	North R	18.1	234.2	7.3	11.1	3.7	23.5	5.9	10.2	49.3	6.8	51.7	12.3	148.6	2232.9	2271.9	4.2
JL-10	North R	18.9	164.8	7.0	7.4	3.6	16.9	3.2	8.3	23.1	7.7	34.9	11.8	99.4	1516.4	1410.3	7.0
JL-11	Xinan R	13.3	131.3	7.5	2.5	2.1	19.3	2.6	2.0	4.5	3.2	61.9	14.1	81.2	1337.9	1216.1	9.1
JL-12	North R	15.3	183.5	7.0	12.7	4.0	17.1	3.2	7.1	34.6	8.2	34.1	12.4	116.1	1761.3	1611.5	8.5
JL-13	Xiazhe R	15.9	49	6.7	2.9	2.6	4.4	0.5	1.6	3.7	4.7	15.4	14.0	42.0	448.8	449.7	-0.2
JL-14	North R	13.9	193.8	6.9	10.9	4.7	18.1	3.4	7.1	36.1	10.6	32.2	11.1	118.2	1772.3	1652.3	6.8
JL-15	North R	18.1	165.4	8.3	8.6	4.3	22.3	3.2	8.6	29.7	11.3	35.9	8.7	114.7	1861.5	1632.9	12.3
JL-16	Xiandu R	16.9	82.4	7.4	5.6	2.9	7.0	1.4	2.5	3.8	11.2	23.0	21.3	67.1	777.7	707.4	9.0
JL-17	North R	14.8	186.7	7.1	9.4	5.0	18.3	3.0	7.3	31.0	11.4	33.3	10.7	112.8	1700.7	1580.7	7.1
JL-18	North R	17.6	128.1	7.0	7.4	4.3	15.3	2.3	5.4	22.1	10.2	29.2	11.6	93.2	1385.9	1256.5	9.3
JL-19	North R	15.4	131	7.1	8.0	4.5	16.2	2.5	6.0	24.5	10.6	28.8	11.1	97.7	1474.1	1322.3	10.3
JL-20	North R	15.4	125.9	7.1	7.9	4.1	15.7	2.4	6.3	23.7	10.3	28.3	11.8	96.3	1427.9	1301.1	8.9
JL-21	Longjing R	22.7	151.6	8.8	10.2	5.5	13.6	2.6	9.1	8.4	14.7	38.9	18.9	102.4	1477.2	1305.0	11.7
JL-22	North R	18.6	155.4	7.5	9.9	4.6	17.5	2.7	9.5	23.9	12.0	31.3	11.8	107.5	1642.4	1474.5	10.2
JL-23	North R	18.4	178.7	7.3	9.2	4.7	18.0	2.8	10.0	24.2	11.9	33.3	11.4	108.7	1649.5	1522.5	7.7
JL-24	Chuanchang R	13.9	103.1	6.9	4.5	3.8	8.2	1.2	2.6	4.2	5.1	39.3	17.1	66.3	797.7	886.5	-11.1
JL-25	Chuanchang R	14.3	141.5	6.9	20.0	4.8	9.3	1.5	22.6	7.1	8.8	34.0	19.7	110.7	1580.7	1482.0	6.2
JL-26	Chuanchang R	15.0	101.3	6.7	5.1	3.6	7.7	1.1	3.1	4.9	12.2	33.6	17.9	72.3	792.0	934.7	-18.0
JL-27	Chuanchang R	16.5	128	6.8	9.6	5.0	12.1	1.8	6.8	10.3	8.0	48.1	19.3	96.9	1297.7	1322.5	-1.9
JL-28	Huashan R	15.4	230.8	7.1	11.2	8.7	19.1	4.1	12.9	17.5	52.0	22.5	20.7	157.3	1995.6	1934.6	3.1
JL-29	West R	16.0	230.8	7.0	13.8	8.2	18.4	3.9	12.9	16.8	44.6	30.6	20.7	154.7	2048.0	1936.4	5.4
JL-30	Longshan R	14.6	173.3	6.9	14.7	5.0	13.5	1.6	9.5	10.7	8.9	55.4	19.3	110.8	1573.3	1540.6	2.1
JL-31	West R	15.7	199.4	7.1	12.2	7.7	16.0	3.0	10.0	16.5	32.2	34.5	18.3	133.0	1771.8	1708.6	3.6
JL-32	West R	19.9	280.9	7.2	19.8	9.2	19.9	3.4	23.6	20.8	20.5	54.5	18.3	162.6	2368.4	2321.3	2.0
JL-33	West R	17.7	253.4	7.1	23.4	10.0	20.4	4.0	28.3	22.7	25.6	48.7	19.0	177.6	2621.7	2479.9	5.4
JL-34	South R	20.1	161.8	7.0	10.6	6.3	14.5	2.9	9.4	7.5	13.1	54.5	27.8	119.4	1581.3	1525.9	3.5
JL-35	South R	20.1	200.1	7.3	17.8	6.6	13.8	3.3	20.4	8.8	16.7	40.7	25.5	133.1	1899.1	1692.9	10.9
JL-36	South R	18.8	295.9	7.4	30.0	8.5	14.8	4.9	42.0	15.1	15.3	43.7	23.0	175.5	2661.9	2464.2	7.4
JL-37	South R	17.1	13628	7.6	3133.5	116.0	102.4	360.7	5543.2	737.7	11.8	81.7	10.5	10056.7	174000	173252	0.4
JL-38	Estuary	16.7	674	7.4	87.9	10.9	22.8	12.1	139.2	39.7	19.2	50.8	15.6	372.7	6232.3	5895.8	5.4
JL-39	Estuary	17.4	7300	7.4	1620.0	58.7	58.6	182.6	2790.7	386.3	14.4	68.6	12.3	5158.0	89892	88122	2.0
JL-40	Estuary	17.0	6956	7.3	1542.7	59.1	54.7	171.9	2694.2	373.0	14.5	67.8	12.3	4956.2	85464	85107	0.4
JL-41	Estuary	17.8	7990	7.3	1742.7	68.2	64.8	206.6	3089.8	424.7	13.7	69.0	12.6	5657.6	97747	97353	0.4
JL-42	Estuary	16.3	1336	7.2	231.0	17.5	28.2	28.6	424.2	83.3	14.3	49.5	13.1	864.8	14251	14740	-3.4

[a] TDS: total dissolved solid. [b] NICB: Normalized Inorganic Charge Balance, NICB = $(TZ^+ - TZ^-) / TZ^- \times 100$.
